# An Improved Cerulean Fluorescent Protein with Enhanced Brightness and Reduced Reversible Photoswitching

**DOI:** 10.1371/journal.pone.0017896

**Published:** 2011-03-29

**Authors:** Michele L. Markwardt, Gert-Jan Kremers, Catherine A. Kraft, Krishanu Ray, Paula J. C. Cranfill, Korey A. Wilson, Richard N. Day, Rebekka M. Wachter, Michael W. Davidson, Megan A. Rizzo

**Affiliations:** 1 Department of Physiology, University of Maryland School of Medicine, Baltimore, Maryland, United States of America; 2 Department of Molecular Physiology and Biophysics, Vanderbilt University, Nashville, Tennessee, United States of America; 3 Center for Fluorescence Spectroscopy and the Department of Biochemistry and Molecular Biology, University of Maryland School of Medicine, Baltimore, Maryland, United States of America; 4 National High Magnetic Field Laboratory and Department of Biological Science, The Florida State University, Tallahassee, Florida, United States of America; 5 Department of Cellular and Integrative Physiology, Indiana University School of Medicine, Indianapolis, Indiana, United States of America; 6 Department of Chemistry and Biochemistry, Arizona State University, Tempe, Arizona United States of America; Cardiff University, United Kingdom

## Abstract

Cyan fluorescent proteins (CFPs), such as Cerulean, are widely used as donor fluorophores in Förster resonance energy transfer (FRET) experiments. Nonetheless, the most widely used variants suffer from drawbacks that include low quantum yields and unstable flurorescence. To improve the fluorescence properties of Cerulean, we used the X-ray structure to rationally target specific amino acids for optimization by site-directed mutagenesis. Optimization of residues in strands 7 and 8 of the β-barrel improved the quantum yield of Cerulean from 0.48 to 0.60. Further optimization by incorporating the wild-type T65S mutation in the chromophore improved the quantum yield to 0.87. This variant, mCerulean3, is 20% brighter and shows greatly reduced fluorescence photoswitching behavior compared to the recently described mTurquoise fluorescent protein in vitro and in living cells. The fluorescence lifetime of mCerulean3 also fits to a single exponential time constant, making mCerulean3 a suitable choice for fluorescence lifetime microscopy experiments. Furthermore, inclusion of mCerulean3 in a fusion protein with mVenus produced FRET ratios with less variance than mTurquoise-containing fusions in living cells. Thus, mCerulean3 is a bright, photostable cyan fluorescent protein which possesses several characteristics that are highly desirable for FRET experiments.

## Introduction

A full complement of colors for genetically-encoded fluorescent proteins has nearly been achieved. Nonetheless, many fluorescent proteins suffer from low brightness and unstable fluorescence that limits their utility for live cell microscopy [1]. Additional disadvantages include properties such as a low quantum yield (QY) [2], inefficient maturation [3], or suboptimal excitation by existing illumination sources [4]–[6]. Thus, there is continued interest in developing fluorescent proteins with properties that are better suited for quantitative microscopy applications.

Among the most widely used fluorescent proteins are those derived from the Aequorea victoria green fluorescent protein (GFP) [7]. The chromophores of fluorescent proteins are formed from three amino acid residues positioned in the interior of the compact β-barrel structure [8]. Spontaneous main chain cyclization of residues 65 and 67 (wild-type GFP) leads to formation of a cyclic α-enolate, that either in its hydrated or dehydrated form, is thought to undergo oxidation to the cyclic imine form. Net elimination of a water molecule and proton abstraction at the β carbon of Tyr^66^ produces a mature chromophore containing a five-membered heterocycle that is fully conjugated to the phenolic group of Tyr^66^ [9]–[12].

Molecular engineering of the chromophore-forming amino acid residues can change both the absorption and emission spectra of the protein, producing blue, cyan and enhanced green fluorescent variants [13]. Replacement of Tyr^66^ with a tryptophan residue introduces the larger indole group into the chromophore π-system, and paradoxically blue-shifts the spectra by ∼30 nm to produce the widely used set of CFPs [14]. Importantly, the spectral properties of fluorescent proteins are determined not only by the chromophore structure alone, but can also be influenced by interactions with the surrounding β-barrel side chains via effects on chromophore orientation, energetics or conformation [15]. For CFPs, the absorption spectra can be approximated by molecular dynamics simulations [16], [17] that have provided a great deal of insight into the experimental observations that side-chain protonation and chromophore conformations can influence both the brightness and absorption spectra of Cerulean [18]. Nonetheless, absorption spectra calculations using these models currently lack the double peak observed experimentally, indicating that a full understanding of the photophysical phenomena underlying the spectral properties of CFPs has not yet been achieved.

Although CFPs are generally dim in comparison to GFPs [1], their blue-shifted fluorescence has made CFPs a popular choice for Förster resonance energy transfer (FRET) experiments when paired with yellow fluorescent proteins (YFPs) such as Citrine [19] or Venus [3]. FRET is typically detected by quantifying changes in sensitized YFP emission, either ratiometrically with CFP fluorescence or using a corrective algorithm. Alternatively, the quenching effect of FRET on CFP fluorescence can be detected by photobleaching YFP or by measuring changes to the CFP fluorescence lifetime. Despite the convenience of the CFP color, the most commonly used CFPs do have drawbacks that limit their utility in FRET experiments. For example, the low QY of commonly used CFPs [20], [21] limits FRET efficiency and the range of energy transfer [22]. Instability of CFP fluorescence can also be problematic for time-resolved FRET experiments. Although corrective measures have been developed to account for photobleaching [23], [24], application of these methods is complicated by the reversibility of the fluorescence loss. This phenomenon is known as reversible photoswitching and has been observed for several fluorescent proteins including Cerulean [25]. Improvements to CFPs that target QY and photostability are thus particularly important for quantitative FRET experiments.

To improve the properties of Cerulean CFP [20] fluorescence, we optimized amino acids in the β-barrel and in the chromophore. The resulting protein is 67% brighter than the original Cerulean fluorescent protein and 21% brighter than the recently reported mTurquoise fluorescent protein [26], which is a high QY CFP derived from the alternative super-folding CFP lineage (SCFP3A) [21]. Although brightness was improved, the absorption and emission spectra of mCerulean3 did not change substantially compared to previous CFPs, including the original Cerulean. mCerulean3 also shows greatly reduced reversible photoswitching, and performs well as a fusion protein. In addition, we show that mCerulean3 provides quantitative advantages for FRET experiments over previous CFP variants.

## Results and Discussion

### Optimization of Cerulean fluorescence

**Figure 1:**
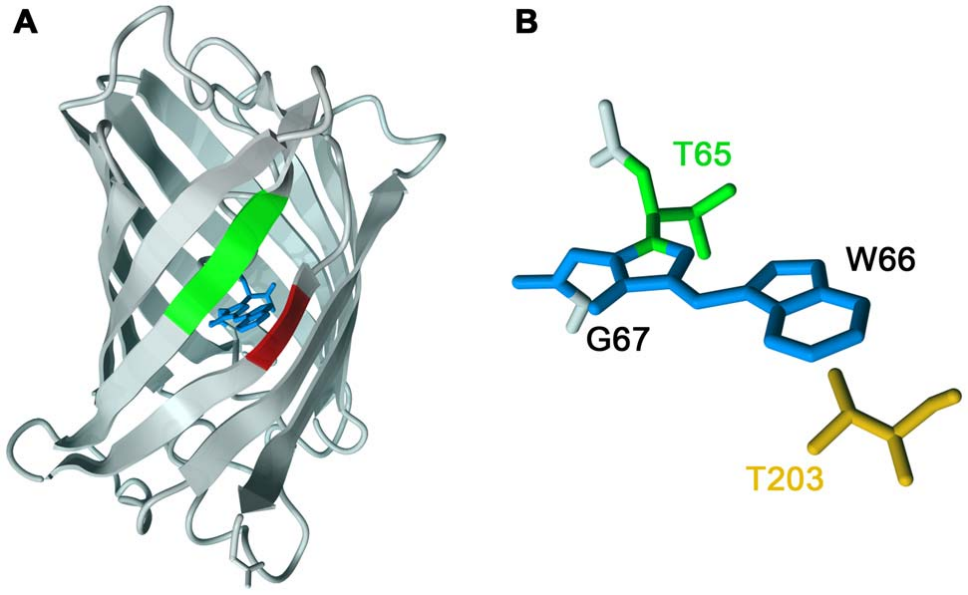
Optimization of Cerulean. A site-directed mutagenesis strategy was employed to optimize Cerulean fluorescence. (A) Residues on β-strand 7 (S147, D148; red), β-strand 8 (L166, I167, R168, H169; green) in the Cerulean X-ray structure (2wso.pdb [27]) were targeted for optimization by site-directed mutagenesis. The chromophore is colored blue. (B) T203 (orange) was targeted for optimization due to its proximity to the chromophore. T65 (green) was also mutated.

**Figure 2:**
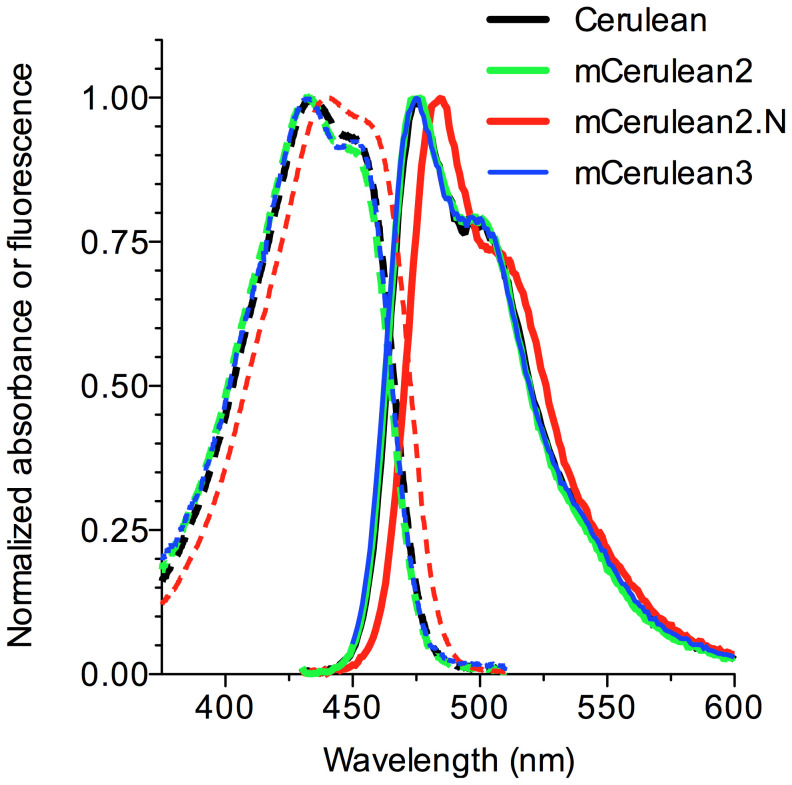
Spectral properties of new CFPs. Absorption (dashed lines) and emission spectra (solid lines) are shown for Cerulean (black), mCerulean2 (green), mCerulean2.N (red), and mCerulean3 (blue). Spectra were normalized to the peak absorption or emission values.

The crystal structure of Cerulean [18], [27] reveals an extended separation between β-strands 7 and 8 (Figure 1A, in red and green, respectively). In general, unfolding fluorescent proteins reduces fluorescence, and direct manipulation of the β-strands by the addition of biosensing domains is known to modulate the molecular brightness of fluorescent proteins [3], [28], [29]. Thus, we hypothesized that optimizing the amino acids that comprise β-strands 7 and 8 might improve the overall brightness of Cerulean. Random mutations were introduced in pairs into the monomeric variant of Cerulean (mCerulean) [5] using degenerate primers (Table S1). Plasmid DNA containing mCerulean mutants were transformed into XL10 bacteria and colony fluorescence was examined by fluorescence microscopy. Plasmids isolated from the brightest colonies were used to generate recombinant proteins for characterization. Although increased colony brightness could result from factors unrelated to protein brightness, such as cell density or differences in protein concentration within the bacterial cells, we did find that increased colony brightness was associated with increased molecular brightness in purified proteins. Mutant CFPs with the highest QYs were selected for additional rounds of optimization. The end product from this series of optimization, mCerulean2, contains 6 mutations (S147H/D148G/K166G/I167L/R168N/H169C; sequence alignment in Figure S1) and is 30% brighter than Cerulean (Table 1) while maintaining similar absorption and emission spectra (Figure 2). Thus, optimization of residues in β-strands 7 and 8 improved the fluorescence of Cerulean.

**Table 1:** Fluorescence properties of CFPs.

Protein	Excitation maximum [nm]	Emission Maximum [nm]	*ε_peak_* (M^−1^cm^−1^)	QY	Brightness^a^	Fluorescence Decay *t_0.5_*^b^ (s)	*k*_fold_^c^ (10^−2^ s^−1^)	*τ*^d^ (ns)(*χ*^2^ e)
Cerulean	434	475	43,000	0.48	21	58	0.54	3.17 (0.03 (2.70)
mCerulean2	432	474	47,000	0.60	28	25	1.62	3.04 (0.03 (4.00)
mCerulean2.N	440	484	49,000	0.48	24	36	1.79	2.63 (0.03 (3.41)
mCerulean2.N(T65S)	439	481	43,000	0.46	20	–	–	–
mCerulean3	433	475	40,000	0.87	35	1100	1.90	4.10 (0.02 (1.05)
mTurquoise	434	474	34,000	0.84	29	61	1.93	4.04 (0.03 (1.04)

a Brightness was calculated as the product of *ε_peak_* and QY.

b *t_0.5_* value of a single exponential fit for fluorescence decay under constant fluorescence illumination at 300 µW/cm^2^.

c Refolding rate from denatured protein was determined using the method from reference [3].

d The fluorescence lifetime time constant (± SD) was obtained from a single-component fit of TCSPC spectroscopy data.

e Value reports the goodness of fit for the lifetime data.

Although the QY of mCerulean2 is 25% greater than Cerulean, it is quite far from the theoretical maximum. Optimization of Thr^203^ (Figure 1B), which is proximal to the chromophore, is known to improve the fluorescence properties of some Aequorea-derived GFP variants [30]. To assess whether optimization of Thr^203^ can enhance the fluorescence properties of mCerulean2, we performed random mutagenesis on this position using degenerate PCR, and screened mutant-containing bacterial colonies for brightness. The brightest protein identified contained the T203I mutation and was named mCerulean2.N. This variant has a ∼9 nm red shift in its fluorescence spectra (Figure 2) which may provide advantages for certain applications since the peak absorption is more closely aligned with 440 nm and 458 nm laser sources compared to mCerulean2. Furthermore, the ∼9 nm shift in the emission spectrum is large enough to permit resolution from the Cerulean spectrum by linear unmixing [31], thus adding another potential color for spectral imaging applications. Nonetheless, the gains in QY observed from the mCerulean2 mutations were negated, even though the molar extinction coefficient was improved. Therefore, mCerulean2.N is a CFP of similar brightness to Cerulean with spectral properties that are more closely aligned with existing laser excitation sources commonly used for fluorescence microscopy.

To further improve mCerulean2 fluorescence, we examined the effect of reverting position Thr^65^ in the chromophore to the wild-type serine residue. Early cyan mutants containing the Ser^65^ had much higher QYs (W2; QY = 0.72) [14] than the widely used S65T-containing ECFP (originally W1B; QY = 0.4) [30]. Furthermore, it has recently been shown that the wild-type T65S substitution can improve the QY of blue fluorescent proteins [2], [32]. Incorporation of T65S into mCerulean2 (mCerulean3) successfully improved the QY of mCerulean2 by 45% and the overall brightness by 25% (Table 1) without changing the absorption or fluorescence emission spectra (Figure 2). The acid stability of mCerulean3 (pKa = 3.2) was also better than mCerulean2 (pKa = 4.8), mCerulean2.N (pKa = 4.5) and mCerulean (pKa = 4.7). In contrast, incorporation of T65S into mCerulean2.N did not improve overall fluorescence, and slightly reduced both the QY and the extinction coefficient. Thus, incorporation of the wild-type Ser^65^ into mCerulean2 greatly improved fluorescence through a mechanism that is incompatible with the T203I mutation, the precise nature of which is unknown.

### Comparison of recombinant mCerulean3 with previous CFPs

We compared the fluorescence properties of the brightest CFP we developed, mCerulean3, with another recently developed CFP that also contains the T65S mutation, mTurquoise [26]. Overall, we found mCerulean3 to be approximately 20% brighter than mTurquoise while the absorption and emission peaks are similar (Table 1). Like mTurquoise, the fluorescence lifetime of mCerulean3 determined by time-correlated single photon counting (TCSPC) spectroscopy fits well to a single exponential component. The maturation times of mTurquoise and mCerulean3 are also very similar (Table 1). Thus, the steady-state spectral qualities of mCerulean3 are roughly equivalent to mTurquoise, with mCerulean3 being the brighter of the two.

**Figure 3:**
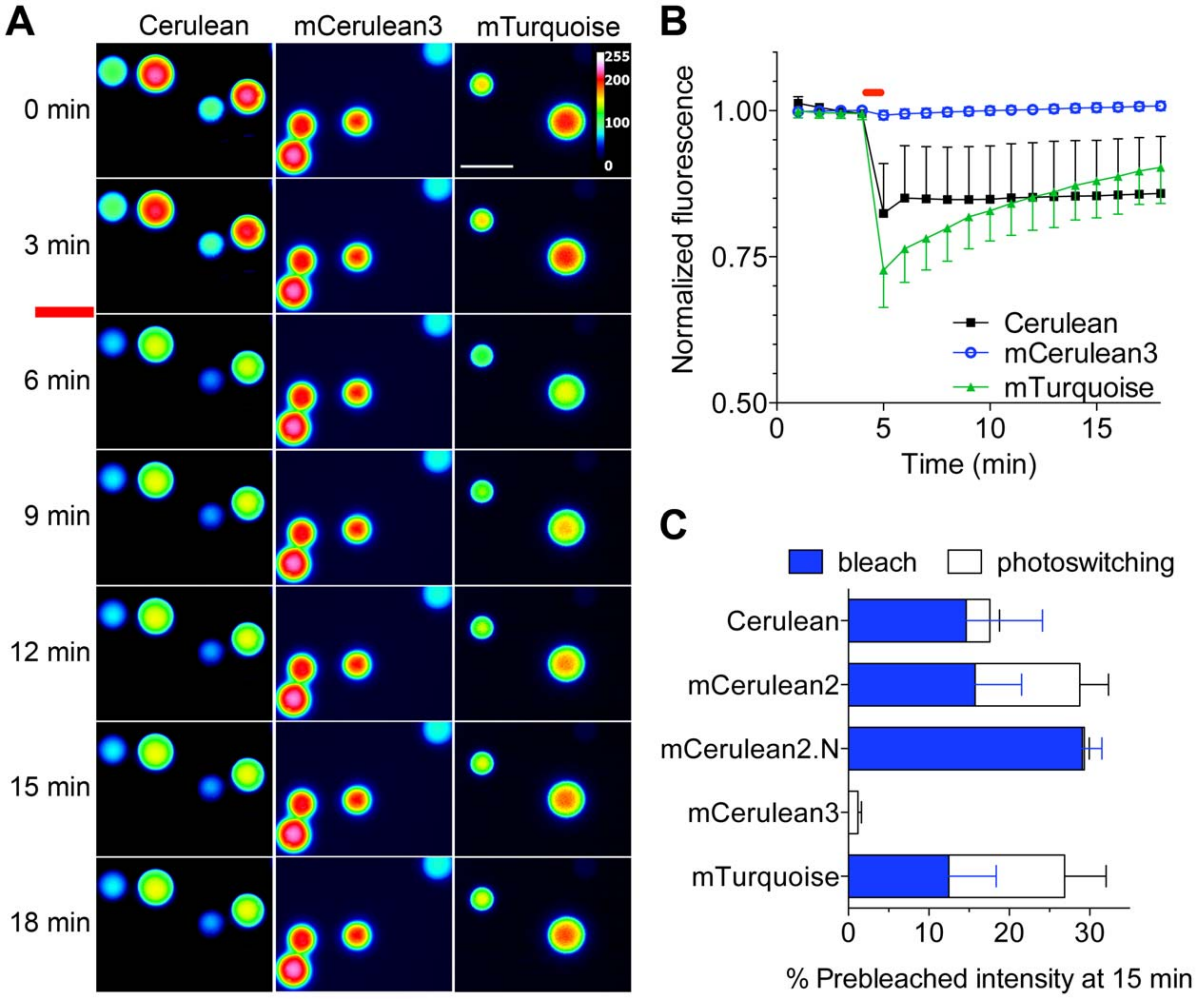
Photostability of recombinant CFPs. Agarose beads labeled with CFPs as indicated were imaged at 60 s intervals under low power illumination (45 µW/cm^2^). At 5 min, the beads were continuously illuminated for 60 s (red bar). (A) Representative images from the experimental data set are shown in pseudocolor to represent bead intensity. The scale bar indicates 10 µm. (B) Bead fluorescence was normalized to prebleached intensity and plotted versus time. Bars indicate SD (n>15 for all samples). (C) The reversible (white) and irreversible (blue) bleached fractions were quantified over the 20 min recovery period.

By convention, we measured the fluorescence decay times for beads labeled with CFPs under continuous illumination. mCerulean2 and mTurquoise behaved similarly to mCerulean; however, mCerulean3 was resistant to fluorescence decay under these conditions. We observed an ∼18-fold longer decay half time for mCerulean3 than mTurquoise. Nonetheless, measurement of fluorescence decay times under continuous illumination has generally not provided reproducible results between laboratories [20], [21], [33], and therefore may not be the most useful predictor of performance. In addition, measurement of the rate of decay under continuous illumination does not take into account that some of the fluorescence loss may be reversible [25] and not indicative of a true photobleach. To distinguish between reversible photoswitching and irreversible bleaching, we imaged beads labeled with recombinant fluorescent proteins at 1 min intervals to establish baseline fluorescence using a low-power illumination intensity that we have successfully used for observation of living cells expressing CFPs. During the imaging protocol we illuminated continuously for a 1 min period to observe the extent of fluorescence decrease, and resumed imaging at 1 min intervals to quantify the extent of reversible photoswitching (Figure 3A, B). For Cerulean, and to a lesser extent, mTurquoise, the reduction of fluorescence induced by the 1 min illumination period was highly variable (for Cerulean, −17.6±8.6% reduction; all values are mean ± standard deviation (SD)) (Figure 3C). In addition, reversible fluorescence photoswitching was observable for both mTurquoise and Cerulean, and accounted for roughly half of the fluorescence decrease observed for mTurquoise beads over the 1 min continuous illumination period. In contrast, mCerulean3 was refractory to fluorescence reduction (1%±0.4, n = 15). Interestingly, we did not observe reversible photoswitching in beads labeled with mCerulean2.N, although the extent of irreversible photobleaching was extensive (Figure 3C). Taken together, we find that recombinant mCerulean3 is exceptionally photostable compared to other Aequorea-derived CFPs.

### Characterization of mCerulean3 expressed in cells

**Figure 4:**
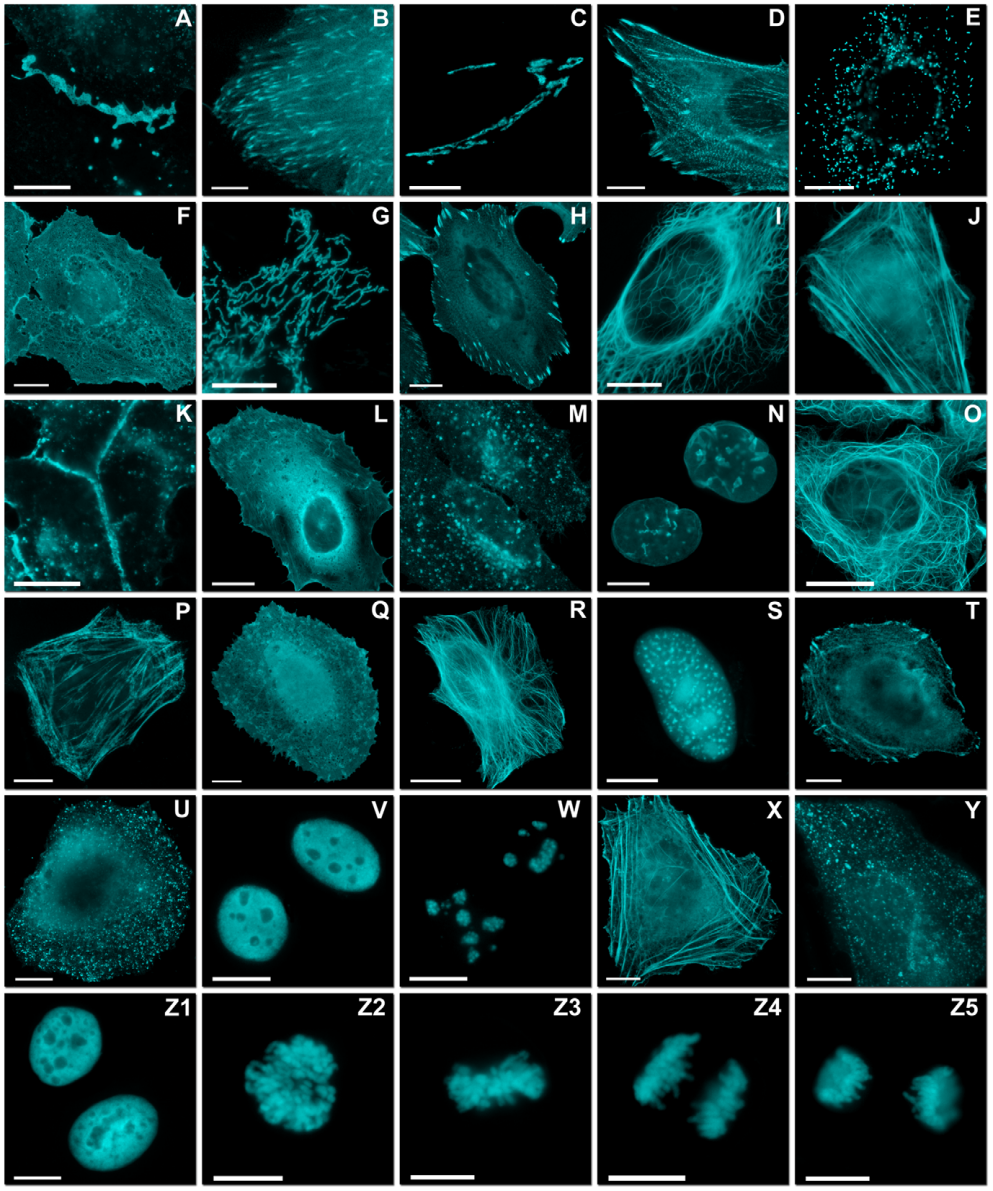
Fluorescence imaging of mCerulean3 fusion vectors. Images were recorded in widefield or laser scanning confocal fluorescence microscopy. (A–M) Fusions to the N-terminus of mCerulean3; for each fusion protein the linker amino acid (aa) length is indicated after the name of the targeted organelle or fusion protein. The origin of the targeting cDNA is indicated in parenthesis. (A) mCerulean3-Cx43-7 (rat); (B) mCerulean3-EB3-7 (human microtubule-associated protein; RP/EB family); (C) mCerulean3-Golgi-7 (N-terminal 81 aa of human β-1,4-galactosyltransferase); (D) mCerulean3-α-actinin (human); (E) mCerulean3-PMP-10 (human peroxisomal membrane protein 2); (F) mCerulean3-c-src-7 (chicken c-src tyrosine kinase); (G) mCerulean3-mitochondria-7 (human cytochrome C oxidase subunit VIII); (H) mCerulean3-zyxin-7 (human); (I) mCerulean3-vimentin-7 (human); (J) mCerulean3-lifeact-7 (N-terminal 17 aa from S. cerevisiae Abp 140); (K) mCerulean3-VE-Cadherin-10 (human vascular epithelial cadherin); (L) mCerulean3-fascin-10 (human fascin); (M) mCerulean3-lysosomes-20 (human lysosomal membrane glycoprotein 1; LAMP-1). (N–Y) Fusions to the C-terminus of mCerulean3. (N) mCerulean3-lamin B1-10 (human); (O) mCerulean-MAP4-10 (mouse microtubule associated protein 4, nucleotides 1918–3135); (P) mCerulean3-lc-myosin-10 (mouse myosin light chain 9); (Q) mCerulean3-CDC42-10 (human cell division cycle 42); (R) mCerulean3-α-tubulin-6 (human); (S) mCerulean3-PCNA-19 (human proliferating cell nuclear antigen); (T) mCerulean3-profilin-10 (mouse profilin); (U) mCerulean3-clathrin light chain-15 (human); (V) mCerulean3-CAF1-10 (mouse chromatin assembly factor 1); (W) mCerulean3-fibrillarin-7 (human fibrillarin); (X) mCerulean3-β-actin-7 (human); (Y) mCerulean-Rab5a-7 (human GTPase Rab5a). (Z1–Z5) mCerulean3-H2B-6 (human) illustrating the various phases of mitosis. (Z1) interphase; (Z2) prophase; (Z3) metaphase; (Z4) anaphase; (Z5) early telophase. Scale bars indicate 10 µm.

To test the suitability of mCerulean3 as a fusion protein, we fused it to a variety of different localization partners, including actin, myosin, and organelle-localized domains (Figure 4). Bright, successfully localized fusions were accomplished using both the N-terminus and C-terminus of mCerulean3, including those that require monomeric character, such as α-tubulin, intermediate filaments, connexin 43, histone H2B, and β-actin. Thus, mCerulean3 is suitable as a fusion partner for a broad range of molecular targets.

**Figure 5:**
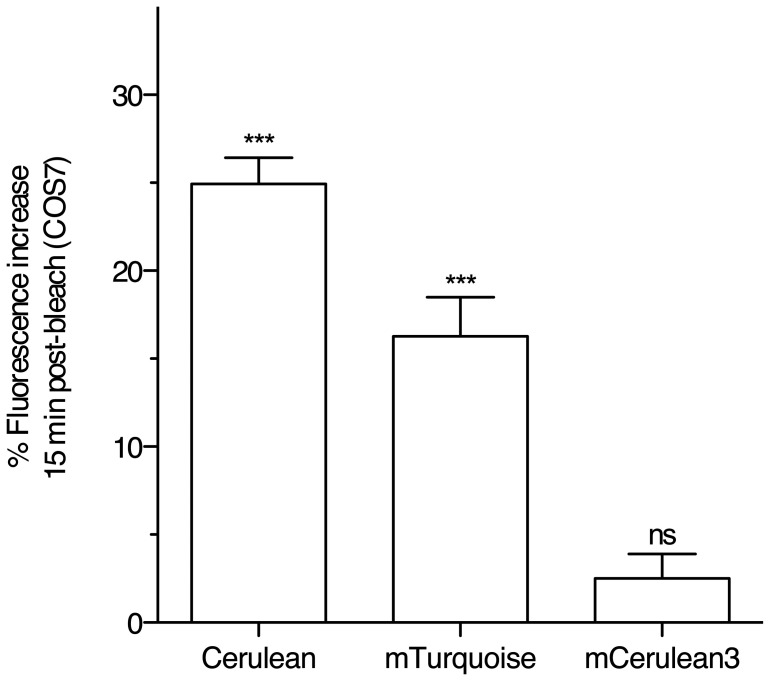
Fluorescence photoswitching behavior of CFPs in living cells. COS7 cells expressing the indicated CFP were examined by widefield microscopy. Cells were bleached to 50% of their initial fluorescence by continuous, high intensity illumination of the full field of view. Recovery of cellular fluorescence was examined 15 min following the bleaching period. Data indicates the mean % recovery of bleached fluorescence after 15 min (n = 20, two-tailed t-test, difference from 0, *** indicates *P*<0.001, mCerulean3 recovery was not statistically significant (ns) under the same test, *P* = 0.09, n = 20).

Although the photostability of existing CFPs is sufficient to enable their widespread use in a great number of applications, reversible photoswitching in living cells has the potential to introduce an undesirable source of error in quantitative applications. To examine the fluorescence photoswitching behavior of CFPs in COS-7 cells, we bleached cells to 50% of their initial fluorescence by continuous illumination of the full field of view over several minutes. Reversible photoswitching was then quantified as the percent increase in fluorescence at 15 min compared to the post-bleach fluorescence intensity (Figure 5). In COS-7 cells, mTurquoise fluorescence recovered to a smaller extent than Cerulean, although the amount of reversible photoswitching was statistically significant (P<0.001, t-test, comparison to 0, n = 20). In contrast, we observed very little reversible photoswitching in cells expressing mCerulean3 (2.5%±6.2, n = 20), and the small amount observed was not statistically significant (t-test, comparison to 0, P>0.05). Thus, mCerulean3 performs well as a fusion protein and displays very little fluorescence photoswitching when expressed in living cells.

### Performance of mCerulean3 in FRET experiments

The CFP:YFP pairing is a widely used combination for FRET experiments in living cells. Consistent with dependence of FRET on the donor QY [22], the calculated Förster distance where half maximal energy transfer occurs (R_0_) was improved for pairing mCerulean3 with the mVenus YFP [3] compared to the monomeric mCerulean alone (Table 2), whereas the Förster distances calculated for mTurquoise and mCerulean3 were very similar. These trends were reflected experimentally in HeLa cells expressing a fused CFP:YFP dimer containing mCerulean, mTurquoise, or mCerulean3 coupled to mVenus. FRET efficiencies were determined in single cell preparations using acceptor photobleaching (Table 2). FRET efficiencies for mTurquoise and mCerulean3-containing fusions were greater than the mCerulean fusion (P<0.001, n = 15, ANOVA, Tukey multiple comparison for mTurquoise:mCerulean, and mCerulean3:mTurquoise); however, FRET efficiencies obtained for mCerulean3 and mTurquoise fusions were not significantly different (P>0.05). These findings are consistent with the calculated R_0_.

**Table 2:** FRET efficiencies of CFP:mVenus pairs.

Protein	mVenus *R_0_* (nm)	*E_FRET_* CFP:YFP dequenching	*E_FRET_* CFP:YFP (FLIM)
mCerulean	5.19	0.24±0.04	0.25±0.02
mTurquoise	5.70	0.30±0.05	0.30±0.03
mCerulean3	5.71	0.33±0.05	0.30±0.01

Accurate FRET efficiencies can be obtained by measuring changes to the donor fluorescence lifetime in the presence of the acceptor [34], and FRET-induced changes to CFP lifetimes can be observed by fluorescence lifetime microscopy (FLIM) [5], [35]. We quantified the FRET efficiencies of our fusion constructs using frequency domain FLIM. Fluorescence lifetimes obtained by FLIM (Figure S2) for mCerulean (τ = 3.00±0.06, n = 10), mCerulean3 (τ = 3.97±0.04, n = 10), and mTurquoise (τ = 4.00±0.05, n = 10) were in fairly good agreement with values obtained for recombinant proteins using time correlated single photon counting (TCSPC) spectroscopy (Table 1). There was more divergence with the frequency-domain FLIM and TCSPC values for Cerulean. This finding is in agreement with previous reports and the divergence is believed to result from the complexity of the Cerulean lifetime [21], [26]. FRET efficiencies obtained with FLIM also agreed with results obtained from donor dequenching (Table 2), with mCerulean3 and mTurquoise-containing fusions showing significantly more FRET than Cerulean (P<0.001, n = 10, ANOVA, Tukey multiple comparison for mTurquoise:mCerulean, and mCerulean3:mTurquoise). As we had observed with donor dequenching, FRET efficiencies for mCerulean3 and mTurquoise containing fusions were not significantly different from each other (P>0.05, n = 10, ANOVA, Tukey multiple comparison test). Taken together with the acceptor photobleaching data, the recently developed high QY CFPs show measurable improvement over Cerulean in the efficiency of CFP:YFP FRET.

**Figure 6:**
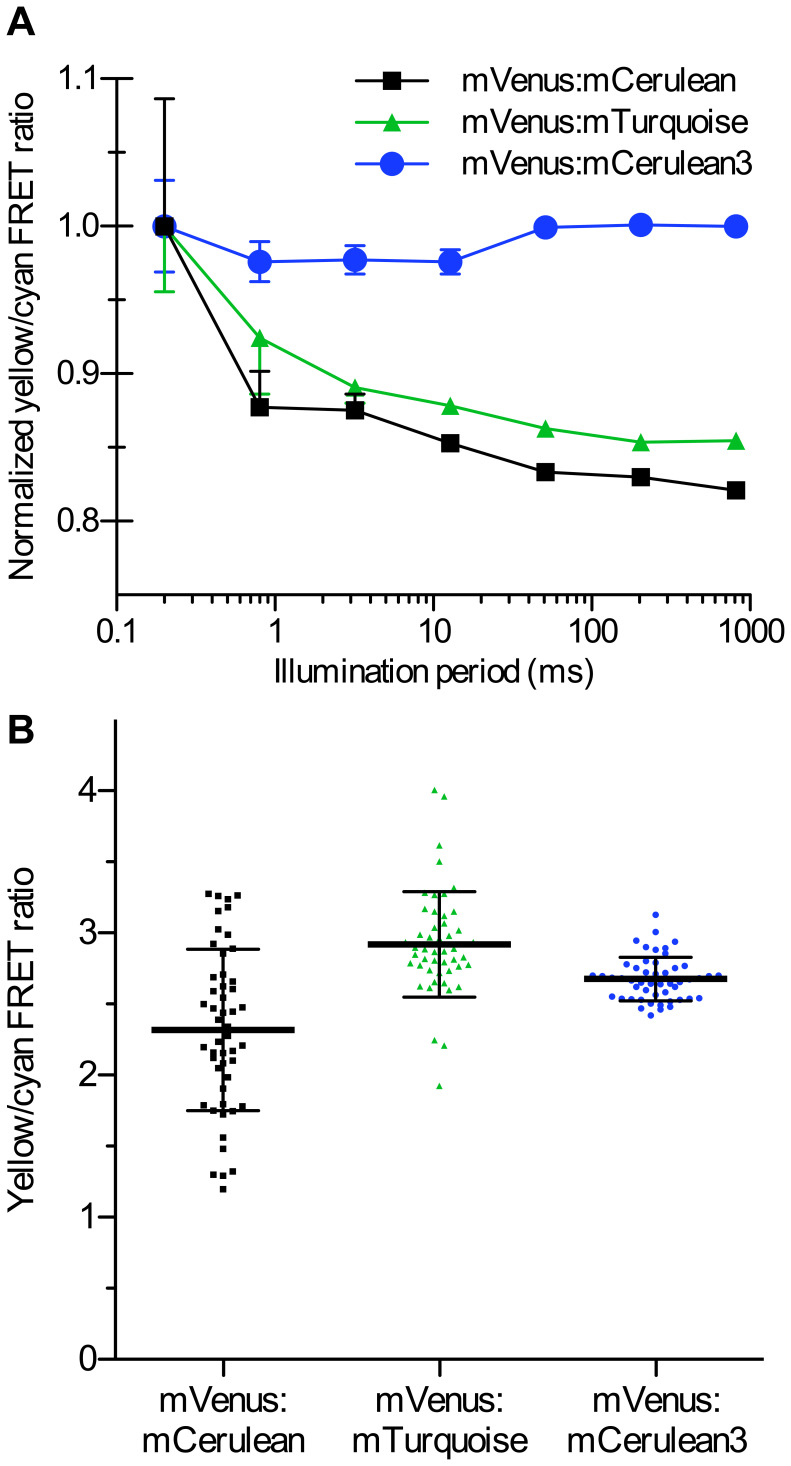
Improved FRET ratio imaging with mCerulean3. (A) To test the dependence of measured FRET ratios on illumination time, agarose beads were labeled with equivalent concentrations of the indicated CFP:mVenus fusion protein. Beads were imaged consecutively using constant illumination intensity (455 nm LED, 600 µW/cm^2^), but a varied illumination period. Cyan and yellow fluorescence were captured simultaneously using an Optical Insights Dual-View containing standard CFP/YFP filter sets. FRET ratios were normalized to the peak FRET ratio. Points indicate the mean and error bars indicate SEM (n = 10). (B) HEK293 cells were transfected with the indicated fusion, and observed by fluorescence microscopy. The yellow/cyan FRET ratio of individual cells is shown (n = 50). Bar indicates the mean, and error bars indicate SD.

Many CFP:YFP FRET experiments utilize the ratio of the donor and acceptor fluorescence to detect changes induced by biosensing of cellular phenomena [36]; however, the instability and decay of CFP fluorescence over time could in theory affect the absolute FRET ratio if the decay in CFP fluorescence is shorter than the image capture time. To examine this in the context of FRET-ratio imaging, we labeled beads with recombinantly-generated CFP:YFP pairs, and examined the FRET ratio of beads over a broad range of image capture times using wide-field microscopy (Figure 6A). FRET ratios from fusions containing mCerulean or mTurquoise and mVenus were 10–20% less at long illumination times (∼1 s) compared with short ones (<1 ms) (Figure 6A). In contrast, FRET ratio measurements performed using mCerulean3 as a donor varied less than 2.5% as exposure time was varied over 4 orders of magnitude. To look at the impact of mCerulean3 photostability on FRET ratios observed in living cells, we expressed the same FRET fusion proteins containing mVenus that we used for the bead preparations in HEK cells (Figure 6B). Using mCerulean as the FRET donor, we observed FRET ratios ranging from 1 to 2.6 (Figure 6B), with a large SD of ±0.57 (n = 50). FRET ratios obtained for mTurquoise fusions were less variable (±0.37, n = 50), but the SD was still 2.5-fold greater than the results obtained with mCerulean3 fusions (±0.15, n = 50). Given that the improvement observed using mCerulean3 over mTurquoise is quite large compared to a small brightness advantage, it is likely that reduced photoswitching is the major component of the reduced variability. Thus, utilization of mCerulean3 as donor protein in FRET experiments reduces variability in ratio measurements.

### Conclusions

Here we report development of a brighter, more photostable Cerulean CFP with very desirable characteristics for quantitative fluorescence microscopy applications. mCerulean3 has very high QY and a single exponential fluorescence lifetime, making it a very useful donor fluorophore for FRET experiments. The reduced photoswitching behavior is also an important advantage for quantitative applications such as FRET and fluorescence recovery after photobleaching. Compared to the other high QY CFP, mTurquoise, FRET with mCerulean3 is similarly efficient, but can be quantified with greater precision. FRET ratios obtained with mCerulean3 display less variance in test preparations and in living cells. Reduced variability in FRET measurements is particularly beneficial for FRET-based biosensors, where the dynamic range is frequently 20% or less [37]–[39]. Furthermore, once mCerulean3 is bleached in cells, the fluorescence does not significantly change through reversible photoswitching. Not only will this simplify interpretation for fluorescence photobleaching experiments, but it also will enhance the utility of corrective photobleaching algorithms [24], [40]. This is of particular importance considering we find that the fraction of fluorescence loss that is reversible can vary greatly, even in highly controlled sample bead preparations. Thus, mCerulean3 possesses special advantages for quantitative live-cell imaging applications.

## Materials and Methods

### Cloning and Gene Construction

Synthetic DNA oligonucleotides for cloning and mutagenesis were purchased from Integrated DNA Technologies (Coralville, IA). PCR products and products of restriction digests were purified by gel electrophoresis and extraction using the QIAquick gel extraction kit (Qiagen, Valencia, CA). Plasmid DNA was purified from overnight cultures using the QIAprep Spin Miniprep kit (Qiagen). Restriction endonucleases were purchased from Invitrogen (Carlsbad, CA) or New England Biolabs (Ipswich, MA). Sequencing was used to confirm the complete cDNA sequences for all fluorescent protein variants and fusion constructs (Florida State University Bioanalytical and Molecular Cloning DNA Sequencing Laboratory, the University of Maryland School of Medicine DNA sequencing facility, GENEWIZ Maryland Laboratory, or the DNA sequencing core facility at the Indiana University School of Medicine). Preparation of DNA for mammalian transfection and transformation was performed using DNA prep kits from Qiagen (QIAprep Spin Miniprep Kit, HiSpeed Plasmid Midi, Plasmid Midi, or Maxi kit).

### Mutagenesis and screening

Mutants were introduced into H_6_mCerulean [5] contained in the pQE9-N1 bacterial expression plasmid [20] by PCR using the Quikchange mutagenesis kit (Agilent Technologies, Wilmington, DE) together with the primers listed in Table S1. Mutant plasmids were transformed into XL10-Gold Ultracompetent cells (Agilent Technologies) according to the manufacturer's instructions. Even though this strain contains the *lacIq* mutation, repression of protein production was sufficiently leaky to enable observation of CFP fluorescence. Transformed cells were plated on LB agar plates containing 100 µg/ml ampicillin, and incubated overnight at 37°C. For screening, 4–15 colonies were transferred to a 25 mm filter (Nucleopore Track-Etch Membrane, GE Healthcare, Carlsbad, CA). The filter was then placed colony side up on the bottom of a sterile 60 mm cell culture dish and screened for brightness (filter set ET436/20X exciter, T455LP beamsplitter, ET480-40m-2p (Chroma Technology Corp., Bellows Falls, VT) using an inverted Zeiss Axiovert 200M with a 1×, 0.025 NA objective lens. Dishes were heated to 50°C to improve contrast. Typically, ∼100 colonies were initially screened for brightness and the 5 brightest colonies were selected for additional screening. Colonies were grown in culture for 1 h at 37°C, and streaked on LB agar plates containing 100 µg/ml ampicillin. The following day, single colonies were transferred to a single filter for comparison, and the brightest colony was selected and cultured in 5 ml LB (100 µg/ml ampicillin) for DNA miniprep (QIAprep spin, Qiagen).

### Purification of recombinant proteins

The Qiagen T5 expression system was used to generate proteins from modified pQE9 vectors described elsewhere [5], [20]. Mutant CFPs selected for additional characterization were transformed into M15(pRep4) bacteria for protein production. Induction with isopropyl β-D-1-thiogalactopyranoside, harvesting and lysis are described elsewhere [5]. Recombinant proteins were purified using Ni^+2^ loaded 1 ml HiTrap Chelating HP columns (GE Healthcare) according to the manufacturer's instructions. Protein concentration was determined using the Advanced Protein Reagent (Sigma-Aldrich, St. Louis, MO), and gel electrophoresis as described previously [5].

### Recombinant protein vectors

H_6_mTurquoise was prepared by site-directed mutagenesis of H_6_SCFP3A using the T65S primers listed in Table S1. Recombinant H_6_mVenus:mCerulean3 contains a 10 aa linker, and was derived using the cloning strategy for the short linker FRET pairs as previously described [5]. Production of H_6_Cerulean and H_6_mCerulean has also been described [5], [20].

### Mammalian expression vectors

For mammalian expression, novel fluorescent protein constructs were subcloned from the pQE9 vector into the pEGFP-C1 using NheI and HindIII restriction sites. N3 and N1 constructs were generated by PCR using previously described methods [5]. To generate subcellular localization fusion vectors used for experiments in Figure 4, the appropriate cloning vector and an mEmerald fusion vector were digested, either sequentially or doubly, with the appropriate enzymes and ligated together after gel purification. Thus, to prepare mCerulean3 N-terminal fusions, the following digests were performed: human non-muscle α-actinin, EcoRI and NotI (vector source, Tom Keller, FSU); human cytochrome C oxidase subunit VIII, BamHI and NotI (mitochondria, Clontech); human zyxin, BamHI and NotI (Clare Waterman-Storer, NIH); rat α-1 connexin-43, EcoRI and BamHI (Matthias Falk, Lehigh University); human H2B, BamHI and NotI (George Patterson, NIH); N-terminal 81 aa of human β-1,4-galactosyltransferase, BamHI and NotI (Golgi, Clontech); human microtubule-associated protein EB3, BamHI and NotI (Lynne Cassimeris, Lehigh University); human vimentin, BamHI and NotI (Robert Goldman, Northwestern University); human peroxisomal membrane protein 2, NotI and AgeI (peroxisomes; OriGene); c-src, BamHI and NotI (chicken c-src tyrosine kinase, Marilyn Resh, Sloan-Kettering Institute); lifeact, BamHI and NotI (N-terminal 17 aa from S. cerevisiae Abp 140, IDT); VE-cadherin, BamHI and NotI (human vascular epithelial cadherin, Andreea Trache, Texas A&M); fascin, BamHI and NotI (human fascin, OriGene). To prepare mCerulean3 C-terminal fusions, the following digests were performed: human β-actin, NheI and BglII (Clontech); human α-tubulin, NheI and BglII (Clontech); human light chain clathrin, NheI and BglII (George Patterson, NIH); human lamin B1, NheI and BglII (George Patterson, NIH); mouse MAP4, NheI and BglII (mouse microtubule associated protein 4, nucleotides 1918–3135, Richard Cyr, Penn State University); mouse light chain 9 myosin, NheI and BglII (Patricia Wadsworth, University of Massachusetts); human CDC-42, NheI and BglII (OriGene); PCNA, AgeI and BspEI (David Gilbert, FSU); mouse CAF-1, NheI and BglII (Akash Gunjan, FSU); human fibrillarin, AgeI and BspEI (Dimitry Chudakov, Russian Academy of Sciences); human GTPase Rab5a, NheI and BglII (Vicky Allen, University of Manchester).

For FRET efficiency and lifetime microscopy experiments, the plasmid encoding the mCerulean:mVenus fusion was provided by Dr. Steven Vogel (NIH) [41], and was used to generate the mCerulean3:mVenus and mTurquoise:mVenus fusion proteins described in Table 2 by substituting the coding sequence for mCerulean with the cDNA for mCerulean3. The mVenus:mCerulean construct used in Figure 6B is the short linker sensor previously described [20]. mVenus:mCerulean3 and mVenus:mTurquoise fusions were constructed using an identical strategy.

### Spectroscopic characterization of recombinant proteins

Absorption spectra were collected using a UV-mini absorbance spectrometer (Shimadzu, Columbia, MD), and emission spectra were collected on QM-3 fluorometer (Photon Technology International, Birmingham, NJ). Molar extinction coefficients were calculated as previously described [5]. Förster distances were calculated as previously described [5] using QY, extinction coefficients, and overlap integrals generated from freshly prepared mCerulean, mTurquoise, mCerulean3, and mVenus proteins. The pKa for fluorescence was measured and calculated also as described [20]. Renaturation assays were performed by a previously described method [3]. For the initial comparative QY measurements, optical densities at 425 nm were set to 0.05, and total fluorescence was measured from 430 to 600 nm (425 nm excitation). Polarizers were set at magic angle conditions to account for polarization bias. Final data normalization was performed using a fluorescein solution as a reference standard (QY = 0.95 in 0.1 M NaOH [42]; 425 nm excitation, 430–650 nm emitted light collection), using identical instrument parameters for CFP specimens, including the expanded emission range. Fluorescence lifetimes were obtained by TCSPC spectroscopy using a FluoTime 100 (PicoQuant Photonics, Westfield, MA). Data was acquired under magic angle conditions using 440 nm excitation, and collection with a 475 nm long pass filter. Time constants were obtained from a single component fit using FluoFit software (PicoQuant).

### Experiments using fluorescent-protein labeled beads

For bleaching measurements, HiTrap beads were labeled with fluorescent protein as previously described [43], [44] and mounted in Prolong Gold (Invitrogen). Imaging was performed using an AxioObserver microscope platform (Carl Zeiss MicroImaging, Thornwood, NY). High-speed imaging of fluorescence decay under constant 455 nm light-emitting diode illumination was performed using a water-cooled C9100-13 EM-CCD (Hamamatsu, Bridgewater, NJ) to capture 300 images. Illumination power was measured at the objective lens prior to experimentation using a Newport 1918C power meter. Decay in fluorescence was quantified and fit to a single exponential decay using Prism software (Graphpad Software, La Jolla, CA). The *t_0.5_* for the curve fit was reported. Photoswitching measurements were performed using a water-cooled Hamamatsu C10600 Orca-R2 CCD, under 455 nm light-emitting diode illumination. Cyan fluorescence for single color experiments was captured using a High Efficiency CFP filter set (47HE, Zeiss). FRET images were obtained under cyan illumination (455 LED, BP 436/25 filter) and passed through a T455lp dichroic beamsplitter (Chroma). Cyan and yellow fluorescence were simultaneously collected in a single image using an Optical Insights Dual-view system (Photometrics Headquarters, Tucson, AZ) with a CFP/YFP beamsplitter. Images were processed using ImageJ software (http://rsbweb.nih.gov/ij/).

### Cell culture

For imaging experiments, cells were seeded on No 1.5 glass bottom dishes (Mat-Tek Corp., Ashland, MA) prior to transfection. Prior to seeding, COS7 cells and HEK293 cells were cultured in DMEM containing 10% fetal bovine serum (Thermo Fisher Scientific Inc., Waltham, MA). Transfections were performed using FuGENE 6 (Roche Applied Science, Indianapolis, IN), and imaging was performed in phenol-red free Opti-MEM (Invitrogen). HeLa epithelial (CCL-2, ATCC, Manassas, VA) and Grey fox lung fibroblast (CCL-168, ATCC) cells were grown in a 50∶50 mixture of DMEM and Ham's F12 with 12.5% Cosmic calf serum (Thermo Fisher) and transfected with Effectene (Qiagen). For live cell experiments, temperature was maintained at 37°C using a Zeiss incubation system, or a Delta-T culture chamber (Bioptechs, Butler, PA) under a humidified atmosphere of 5% CO2 in air. Mouse pituitary GHFT1 cells [45] for FLIM experiments (Table 2, Figure S2) were maintained as monolayer cultures in DMEM containing 10% newborn calf serum. Plasmid DNA was introduced by electroporation as described earlier [6]. The amount of DNA was kept constant for each electroporation using empty vector DNA. Cells were then transferred to Nunc Lab-TekII chambered coverglass (Thermo Fisher), and maintained in an incubator overnight before imaging. The coverglass with attached cells was rinsed, and the chambers filled with CO_2_-independent medium and placed on the microscope stage.

### Live cell imaging experiments

Imaging experiments in Figure 4 were performed with a Nikon TE-2000 inverted microscope equipped with QuantaMax™ filters (Omega Optical, Brattleboro, VT) and a Cascade II camera (Roper Scientific, Trenton, NJ), or an IX71 microscope (Olympus America, Center Valley, PA) equipped with BrightLine™ filters (Semrock, Rochester, NY) and a Hamamatsu ImagEM™ camera. Laser scanning confocal microscopy was conducted on a C1Si (Nikon) and an Olympus FV1000, both equipped with argon-ion 457 nm and 405-nm diode lasers and proprietary filter sets. Spinning disk confocal microscopy was performed on an Olympus DSU-IX81 equipped with a Lumen 200 illuminator (Prior Scientific, Rockland, MA), a Hamamatsu 9100-12 EMCCD camera, Semrock filters, and 10-position filter wheels driven by a Lambda 10-3 controller (Sutter Instrument Company, Novato, CA). In some cases, cell cultures expressing CFP fusions were fixed before imaging in 2% paraformaldehyde (Electron Microscopy Sciences, Hatfield, PA 19440) and washed several times in PBS containing 0.05 M glycine before mounting with a polyvinyl alcohol-based medium. Morphological features in all fusion constructs were confirmed by imaging fixed cell preparations on coverslips using a Nikon 80i upright microscope and Omega ECFP filter set (XF144-2) coupled to a Hamamatsu Orca ER or a Photometrics CoolSNAP™ HQ2 camera.

Imaging experiments on COS7 cells in Figure 5 were performed using a 40×, 1.3 NA Plan-NeoFluar oil objective (Zeiss), and illuminated with a 455 nm light emitting diode illuminator filtered through a high efficiency CFP filter set (436/20 exciter, T455LP beamsplitter, 480/40 emitter; Zeiss) and collected with a water-cooled Hamamatsu C9100-13 EM CCD. Illumination intensity and collection speeds were held constant across samples.

Acceptor photobleaching measurements were performed using confocal microscopy. Data was collected 25 frames post bleaching to minimize the impact of mVenus photoconversion, (photoconverted mVenus was 0.17±0.09 at frame 1, –0.01±0.06 at frame 25, n = 11). FRET Imaging experiments on HEK293 cells in Figure 6 were performed using a 40×, 0.95 NA Plan-ApoChromat objective (Zeiss) with CFP excitation and FRET image collection as described above for the bead calibration.

Fluorescence lifetime measurements were made using the phasor FLIM method recently described [46]–[48]. Images were collected using an Olympus IX71 epi-fluorescent microscope equipped with a U Plan S-APO 60× 1.2 NA water objective lens. The microscope was coupled to the FastFLIM frequency domain system (ISS, Champaign, IL) and uses a 0.5 mW 448 nm diode laser modulated at a fundamental frequency of 20 MHz for excitation of mCerulean. The lifetime images were acquired using a 480/40 nm emission filter, and a typical data acquisition time of ∼20 s resulted in photon counts sufficient for high confidence determination of fluorescence lifetimes. Phasor plots (Figure S2) show the entire distribution of the mCerulean and mCerulean3 fluorescence lifetimes in the image. The phasor transformation does not assume any fitting model for fluorescence lifetime decays, but rather expresses the overall decay in each pixel in terms of the polar coordinates on a universal semi-circle [49]. Once the lifetimes of the unquenched donors were determined, the FRET efficiency (*E_FRET_*) was calculated by the equation: 
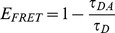
where *τ_D_* is the lifetime of the donor in the absence of the acceptor, and *τ_DA_* is the lifetime of the donor in the presence of the acceptor.

### Statistical Analysis

The indicated statistical tests were performed using Prism software.

## Supporting Information

Figure S1 **Amino acid sequence alignment of CFPs.** An alignment of the amino acid sequences for CFPs created during optimization is shown with the original mCerulean sequence and also the sequence of mTurquoise. Amino acid substitutions are highlighted in red. By convention, the amino acid are referred to by their position in WT Aequorea GFP, which excludes the Val insertion at position 2. Thus, Thr^65^ is actually is the sixty-sixth amino acid in the mCerulean sequence.
(TIF)

Figure S2 **Fluorescence lifetime microscopy of mCerulean3.** Fluorescence lifetime images of mouse pituitary GHFT1 cells expressing mCerulean (left panel), mCerulean3 (right panel). Images were obtained using the frequency domain method. The bottom panels show polar plot analyses of the lifetime distributions for each image using the first harmonic (20 MHz), calculated by the method of Redford and Clegg [49]. The average lifetime was determined for each region of interest (red squares) and the scale bars indicate 10 µm.
(TIF)

Table S1 Characteristics of intermediary mCerulean variants and the primers used in their development.
(DOC)
