# Supplementary figures and images for: An Improved Cerulean Fluorescent Protein with Enhanced Brightness and Reduced Reversible Photoswitching
Source: PLoS One. 2011 Mar 29;6(3):e17896. doi: 10.1371/journal.pone.0017896 (PMC3066204; doi:10.1371/journal.pone.0017896)

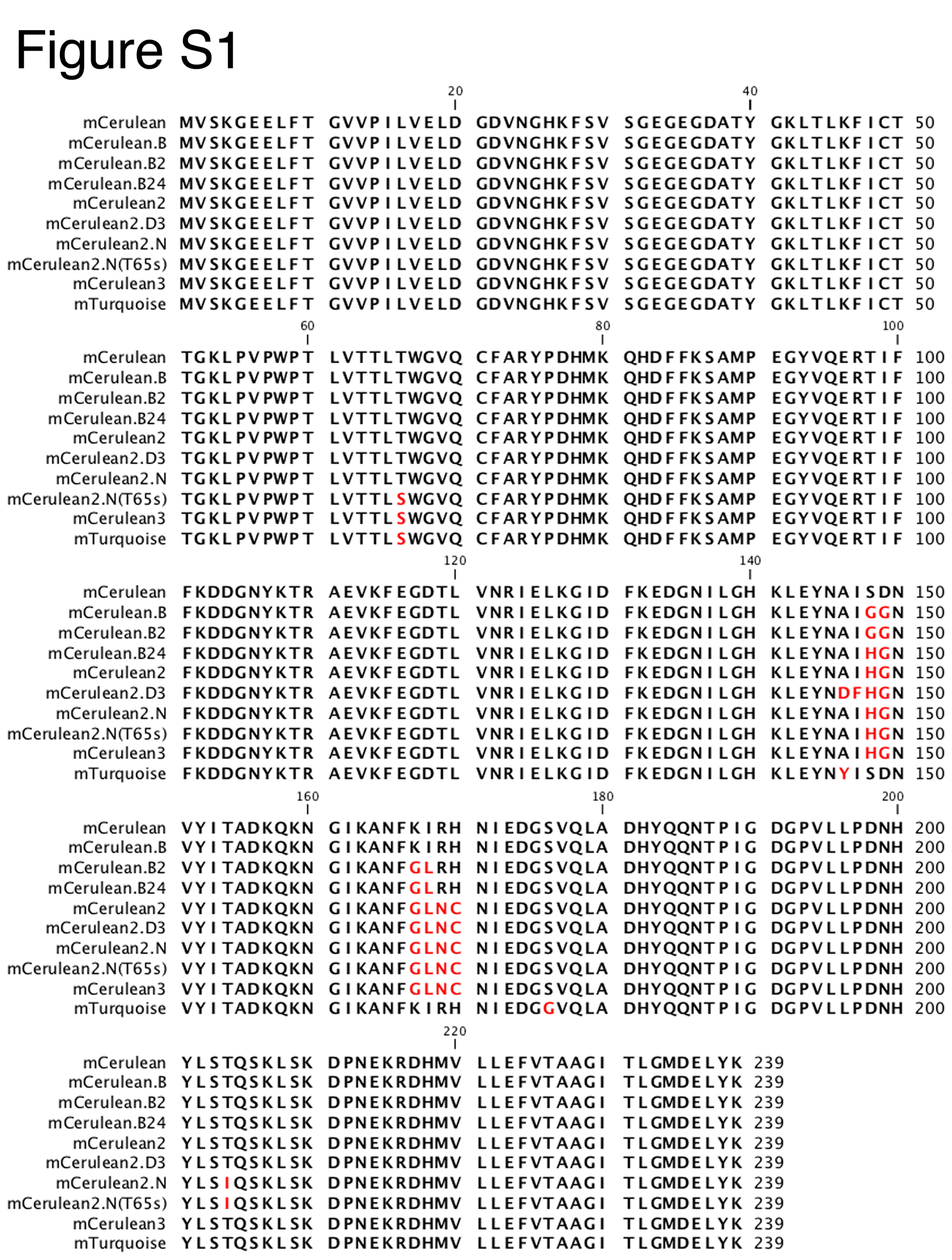

Supplement: Figure S1 — Amino acid sequence alignment of CFPs. An alignment of the amino acid sequences for CFPs created during optimization is shown with the original mCerulean sequence and also the sequence of mTurquoise. Amino acid substitutions are highlighted in red. By convention, the amino acid are referred to by their position in WT Aequorea GFP, which excludes the Val insertion at position 2. Thus, Thr65 is actually is the sixty-sixth amino acid in the mCerulean sequence. (TIF) [file pone.0017896.s001.tif]

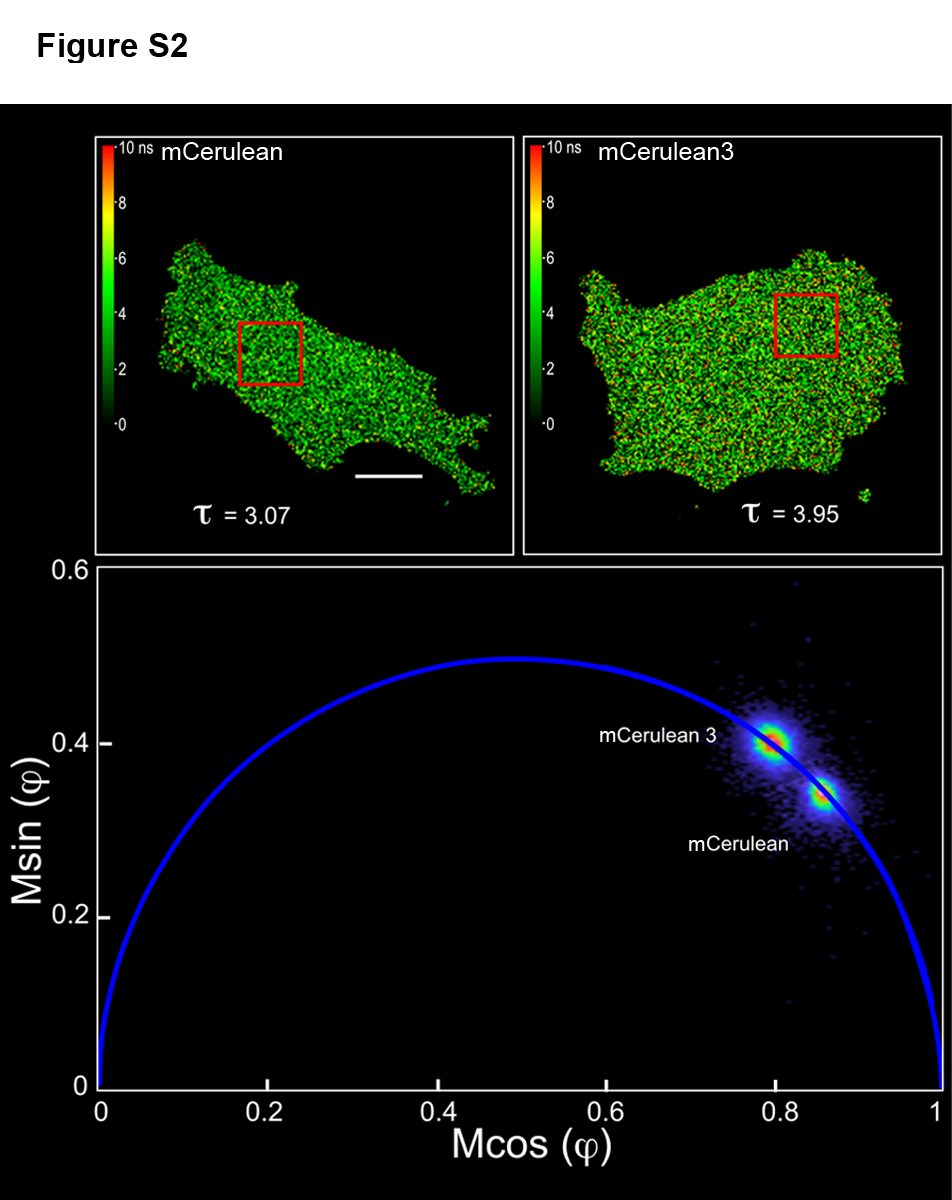

Supplement: Figure S2 — Fluorescence lifetime microscopy of mCerulean3. Fluorescence lifetime images of mouse pituitary GHFT1 cells expressing mCerulean (left panel), mCerulean3 (right panel). Images were obtained using the frequency domain method. The bottom panels show polar plot analyses of the lifetime distributions for each image using the first harmonic (20 MHz), calculated by the method of Redford and Clegg [49]. The average lifetime was determined for each region of interest (red squares) and the scale bars indicate 10 µm. (TIF) [file pone.0017896.s002.tif]
