# Supplementary material for: An Improved Cerulean Fluorescent Protein with Enhanced Brightness and Reduced Reversible Photoswitching
Source: PLoS One. 2011 Mar 29;6(3):e17896. doi: 10.1371/journal.pone.0017896 (PMC3066204; doi:10.1371/journal.pone.0017896)
Supplement: Table S1 — Characteristics of intermediary mCerulean variants and the primers used in their development. (DOC) [file pone.0017896.s003.doc]

**Table S1** Characteristics of intermediary mCerulean variants and the primers used in their development.

| **Protein** | **Introduced**  **Mutations** | ***λ*_ab_^a^(*ε*^b^**) | ***λ*_em_^c^(QY)** | **Brightness^d^** | **Primers** |
| --- | --- | --- | --- | --- | --- |
| mCerulean |  | 434(43) | 475(0.48) | 21 |  |
| mCerulean.B | S147G, D148G | 434(43) | 473(0.49) | 21 | S 5'-ctggagtacaacgccatcNNcNNcaacgtctatatcaccgc-3'  A 5'-gcggtgatatagacgttgNNgNNgatggcgttgtactccag-3' |
| mCerulean.B2 | K166G, I167L | 432(39) | 471(0.50) | 20 | S 5'-cggcatcaaggccaacttcNNNNNccgccacaacatcgaggac-3'  A 5'-gtcctcgatgttgtggcggNNNNNgaagttggccttgatgccg-3' |
| mCerulean.B24 | G147H | 433(43) | 473(0.52) | 22 | S 5'-ctggagtacaacgccatcNNcNNcaacgtctatatcaccgc-3'  A 5'-gcggtgatatagacgttgNNgNNgatggcgttgtactccag-3' |
| mCerulean2 | R168N, H169C | 432(47) | 474(0.60) | 28 | S 5’-ggccaacttcggcctcNNcNNcaacatcgaggacggc-3’  A 5’-gccgtcctcgatgttgNNgNNgaggccgaagttggcc-3’ |
| mCerulean2.D3 | A145D, I146F | 434(45) | 472(0.52) | 23 | S 5’-cacaagctggagtacaacNNcNNccacggccgcgcctata-3’  A 5’-tataggcgcggccgtggNNgNNgttgtactccagcttgtg-3’ |
| mCerulean3 | T65S | 433(40) | 475(0.87) | 35 | S 5’-gaccaccctgagctggggcgtgc-3’  A 5’-gcacgccccagctcagggtggtc-3’ |
| mCerulean2.N | T203I | 440(49) | 484(0.48) | 24 | S 5’-caaccactacctgagcNNNcagtccaagctgagcaa-3’  A 5’-ttgctcagcttggactgNNNgctcaggtagtggttg-3’ |
| mCerulean2.N.65 | T203I, T65S | 439(43) | 481(0.46) | 20 | S 5’-gaccaccctgagctggggcgtgc-3’  A 5’-gcacgccccagctcagggtggtc-3’ |

^a^Peak absorbance wavelength (nm). ^b^Peak molecular extinction coefficient (10^3^ M^-1^cm^-1^). ^c^peak emission wavelength (nm). ^d^Molecular brightness was calculated as the product of the absorbance and the quantum yield(QY). N=A,T,C, or G, S=sense primer and A=antisense primer.
